# A newly discovered *Bordetella* species carries a transcriptionally active CRISPR-Cas with a small Cas9 endonuclease

**DOI:** 10.1186/s12864-015-2028-9

**Published:** 2015-10-26

**Authors:** Yury V. Ivanov, Nikki Shariat, Karen B. Register, Bodo Linz, Israel Rivera, Kai Hu, Edward G. Dudley, Eric T. Harvill

**Affiliations:** Department of Veterinary and Biomedical Sciences, Center for Infectious Disease Dynamics, Center for Molecular Immunology and Infectious Diseases, Pennsylvania State University, University Park, W213 Millennium Science Complex, University Park, PA 16802 USA; Department of Food Science, Center for Infectious Disease Dynamics, Center for Molecular Immunology and Infectious Diseases, Pennsylvania State University, University Park, PA 16802 USA; Present address: Department of Biology, Gettysburg College, Gettysburg, PA 17325 USA; USDA, Agricultural Research Service, National Animal Disease Center, Ames, IA 50010 USA; Lee Kong Chian School of Medicine and Singapore Centre on Environmental Life Sciences Engineering, Nanyang Technological University, Singapore, 637551 Singapore

**Keywords:** *Bordetella pseudohinzii*, Type II CRISPR, Cas9, SpyCas9, Bacteria, Genome editing, Protospacer, GC-content, HGT

## Abstract

**Background:**

Clustered regularly interspaced short palindromic repeats (CRISPR) and CRISPR-associated genes (*cas*) are widely distributed among bacteria. These systems provide adaptive immunity against mobile genetic elements specified by the spacer sequences stored within the CRISPR.

**Methods:**

The CRISPR-Cas system has been identified using Basic Local Alignment Search Tool (BLAST) against other sequenced and annotated genomes and confirmed via CRISPRfinder program. Using Polymerase Chain Reactions (PCR) and Sanger DNA sequencing, we discovered CRISPRs in additional bacterial isolates of the same species of *Bordetella*. Transcriptional activity and processing of the CRISPR have been assessed via RT-PCR.

**Results:**

Here we describe a novel Type II-C CRISPR and its associated genes—*cas1*, *cas2,* and *cas9*—in several isolates of a newly discovered *Bordetella* species. The CRISPR-*cas* locus, which is absent in all other *Bordetella* species, has a significantly lower GC-content than the genome-wide average, suggesting acquisition of this locus via horizontal gene transfer from a currently unknown source. The CRISPR array is transcribed and processed into mature CRISPR RNAs (crRNA), some of which have homology to prophages found in closely related species *B. hinzii*.

**Conclusions:**

Expression of the CRISPR-Cas system and processing of crRNAs with perfect homology to prophages present in closely related species, but absent in that containing this CRISPR-Cas system, suggest it provides protection against phage predation. The 3,117-bp *cas9* endonuclease gene from this novel CRISPR-Cas system is 990 bp smaller than that of *Streptococcus pyogenes*, the 4,017-bp allele currently used for genome editing, and which may make it a useful tool in various CRISPR-Cas technologies.

**Electronic supplementary material:**

The online version of this article (doi:10.1186/s12864-015-2028-9) contains supplementary material, which is available to authorized users.

## Background

*C*lustered *r*egularly *i*nterspaced *s*hort *p*alindromic *r*epeats (CRISPR)-Cas (*C*RISPR-*as*sociated) systems serve as an adaptive immune mechanism in prokaryotes that confer protection against bacteriophages and other mobile elements and vectors [1]. A typical CRISPR-*cas* locus includes a CRISPR array of containing direct repeats (DR) separated by spacers (Sp) and adjacent *cas* genes [2]. In response to invading DNA, CRISPRs acquire short fragments of the foreign nucleic acid sequences and insert those as new spacers at the beginning of the CRISPR array, with each spacer flanked on both sides by direct repeat sequences. This acquisition step involves Cas1, Cas2, and Cas9 proteins [3–5]. Cas9, the signature of Type II CRISPR systems [6], is a RNA-guided endonuclease. CRISPR arrays are transcribed and subsequently processed into small individual *CR*ISPR *RNA*s (crRNA). This “maturation” step of the array precursor requires a *trans*-activating crRNA (tracrRNA), an endogenous ribonuclease RNase III, and Cas9 [7, 8]; although RNase-III-independent systems exist for some bacteria with Type II-C CRISPRs [9]. In *Streptococcus pyogenes,* one of the most well studied Type II CRISPR-Cas systems, both tracrRNA and crRNA guide the Cas9 endonuclease to a complementary target sequence (protospacer) to mediate a double-stranded DNA break during target interference. For additional specificity and to avoid cutting within the array itself (autoimmunity), RNA-guided Cas9 cleavage requires a protospacer adjacent motif (PAM; in *S. pyogenes*: 5′-NGG-3′) flanking the target site. The specifically targeted endonuclease activity of the *S. pyogenes* Type II-A CRISPR-Cas system has allowed for important breakthrough applications in RNA-guided control of gene expression, genome engineering, and genome editing of multiple organisms [7, 10]. But limitations of this particular system have led to a search for new CRISPR-Cas systems with altered features.

The publicly available CRISPRfinder program [11] identified CRISPR-*cas* loci in 45 % (1176/2612) of the bacterial genomes analyzed, but CRISPR-Cas systems have not been identified within the genus *Bordetella*. This genus, which is comprised of nine species, is historically subdivided into “classical” and “non-classical” bordetellae. The extensively studied classical bordetellae consist of the three respiratory pathogens: *B. pertussis* and *B. parapertussis*, the causative agents of “whooping cough” in humans, and *B. bronchiseptica*, which causes a broad variety of respiratory disease in many different mammals. The non-classical bordetellae are both genotypically and phenotypically different from the classical bordetellae [12]. They consist of the six recently described species: *B. hinzii*, *B. holmesii*, *B. ansorpii*, *B. trematum*, *B. petrii*, and *B. avium*, all of which are only partially characterized [13–17]. While the classical bordetellae are usually associated with respiratory disease, several non-classical species have also been isolated from wound and ear infection, septicemia and endocarditis, predominantly from immunocompromised patients. For example, *B. hinzii*, which is a respiratory pathogen in poultry [18] and rodents [19], has also been isolated from humans with chronic cholangitis [20], bacteremia [21], or fatal septisemia [22].

We set out to define the sequence diversity within the *Bordetella* genus and recently published the genome sequences of numerous isolates from several species [23–26]. During these studies, we discovered a novel species that we named *Bordetella pseudohinzii* (manuscript in preparation). This species is a close relative of *B. hinzii* and naturally infects laboratory-raised mice. *B. hinzii* and *B. pseudohinzii* are distinguishable based on substantial divergence in sequence and gene content, as well as the presence of a CRISPR-Cas system that is unique to the genome of *B. pseudohinzii*. Here, we describe this novel CRISPR-Cas system, demonstrate that it is transcriptionally active and present evidence that it acts as an adaptive immune system against mobile genetic elements, including bacteriophage sequences present in *B. hinzii*. These data suggest that both species have recently shared an ecological niche with phages, which are represented by the prophages in *B. hinzii* genomes and the matching spacers in the genome of *B. pseudohinzii*, and that acquisition of this CRISPR-Cas system protects against those.

## Methods

### Bacterial strains and culture conditions

Bacterial isolates used in this study are described in Additional file 1: Table S1. Cultures used for preparation of DNA were grown at 37 °C on Bordet-Gengou agar containing 10 % sheep’s blood. Stainer-Scholte broth cultures inoculated with colonies from Bordet-Gengou agar and incubated at 37 °C with shaking were used for RNA purification. Growth in broth culture was monitored periodically by checking optical density values (at 600 nm wave length).

### DNA isolation, PCR, and sequencing

DNA used for amplification and sequencing of *cas* genes and the CRISPR array was purified using a commercially available kit (Promega) and was quantified with a Nanodrop 2000 (Thermo Scientific). Primers for amplification of the complete CRISPR array and of the *cas*9, *cas*1, *cas*2, and 16S rRNA genes from other *B. pseudohinzii* isolates (Additional file 2: Table S2) were designed based on the genome sequence of isolate 8-296-03 [GenBank:JHEP01000084]. PCR reactions included 200 μM of dNTPs, 0.5 μM of each primer, 1.5 mM of MgCl_2_, 2 U of Taq polymerase (Roche), 5.0 μl of 10× Buffer II, 10 % DMSO and ~150 ng of purified DNA template in a final volume of 50 μl. Cycling conditions for the amplification of *cas9* were 95 °C for 15 min and 35 cycles of 95 °C for 30 s, 54 °C for 30 s and 72 °C for 3 min, followed by a final elongation step of 72 °C for 4 min. Cycling conditions used with the remaining primer pairs were identical except that the extension time was shortened from 3 min to 1 min. PCR amplicons used for sequencing were purified with ExoSAP-IT (USB Corporation) and sequenced at the National Animal Disease Center Genomics Unit using Applied Biosystems Big Dye Terminator v3.1 on an Applied Biosystems 3130 XL Genetic Analyzer sequencer.

### CRISPR-*cas* locus annotation and protospacer prediction

All *Bordetella* genome sequences available at GenBank were searched for the presence of CRISPR systems using CRISPRfinder [11]. Predicted spacer sequences were submitted to BLAST search to query the nucleotide collection (nr/nt) and whole-genome shotgun contigs (wgs) databases at NCBI. Because *E*-value is inadequate when using short nucleotide sequences as BLAST queries, we introduce a “percent hit quality” score (% HQ) to identify and rank the most significant BLAST hits:

$$ \%HQ=\frac{\%\operatorname{cov}\times \% ID}{100\%}, $$

where *%cov* represents the percentage of coverage between the spacer and predicted protospacer sequences and *%ID* stands for percent nucleotide identity between the two.

### GC-content

The guanine and cytosine content (GC-content) was calculated within a 120-bp sliding window. The difference in GC-content between the CRISPR-*cas* locus and the genome average was determined using a two-proportion test implemented in Minitab 17 (www.minitab.com). Briefly, numbers of G + C (“positive events”) and A + T (“negative events”) were calculated separately for the chromosome [GenBank:JHEP01000084] and for the CRISPR-*cas* locus. Because the CRISPR array consists of repetitive sequences, its GC-content is skewed and, therefore, the array sequence was not included. The significance of the difference (*P*-value) was calculated using two-tailed Fisher’s exact test.

### RNA purification and RT-PCR analysis

**Fig. 1:**
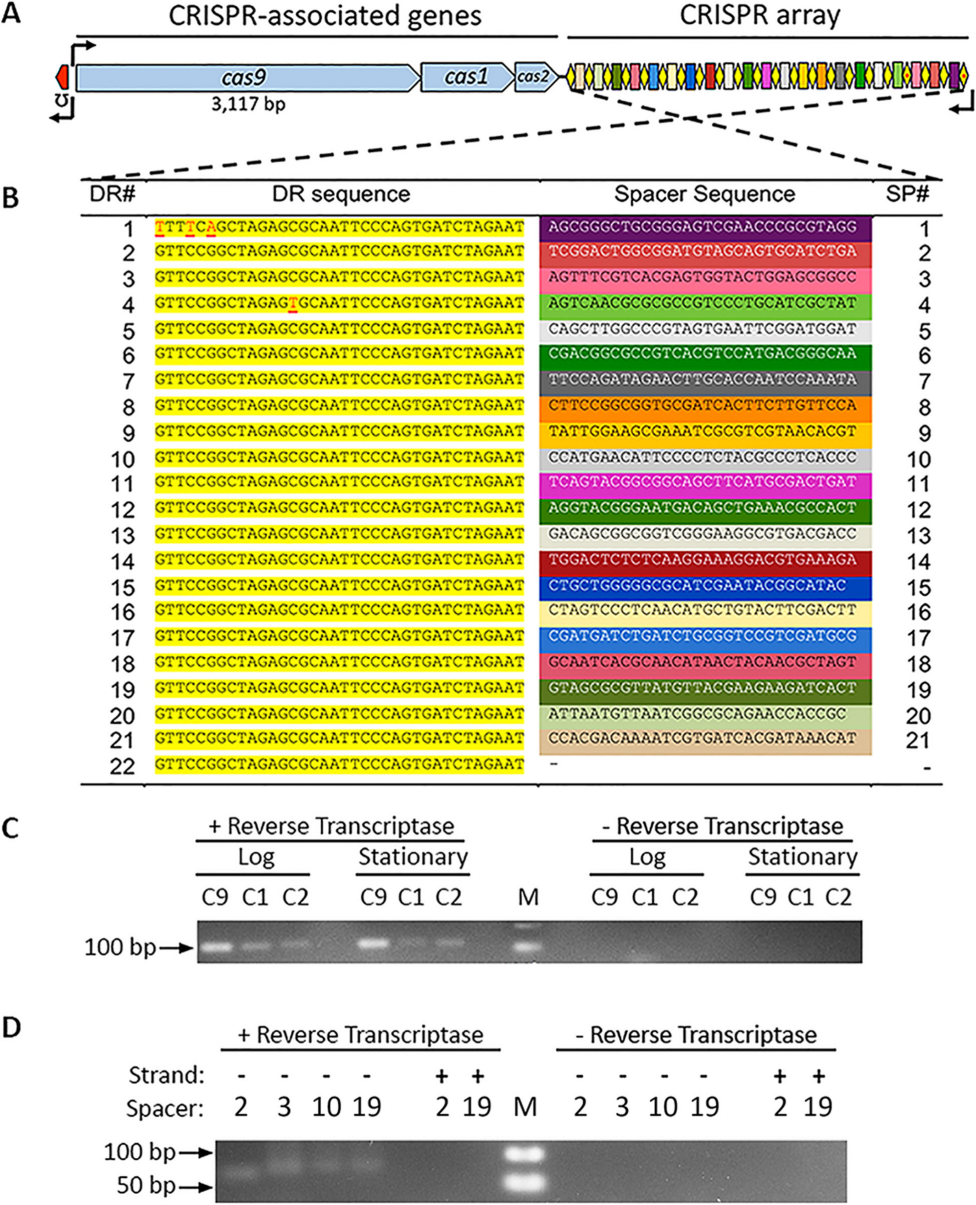
Organization and expression of the Type II-C CRISPR-*cas* locus of *B. pseudohinzii*. **a** Graphical representation of the CRISPR-*cas* locus. The red block upstream of the *cas9* gene is a putative tracrRNA flanked by the predicted promoter (arrow) and stem-loop terminator (up-side-down sigma symbol). The CRISPR array is enlarged relative to the *cas* genes for visual clarity. **b** Nucleotide sequence of the CRISPR array. *SP#* is the spacer sequence number; *DR#* is the direct repeat number. Nucleotides deviating from the DR consensus in DR-1 and DR-4 are highlighted in red. **c** Confirmation of *cas9* (C9), *cas1* (C1), and *cas2* (C2) expression during logarithmic (Log) and stationary phases of growth. Each PCR amplicon was designed to have a similar size. *M*: 100-bp DNA ladder. **d** The CRISPR array is processed into individual, mature crRNAs. Positive and negative strands are relative to the orientation shown in Fig. 1a. *M*: 50-bp DNA ladder

Total RNA was isolated from bacterial cultures during logarithmic growth at OD_600_ = 0.5 and during the stationary phase after overnight growth using the TRIzol® Plus RNA Purification System (Life Technologies). To eliminate any residual DNA in the samples, a DNase treatment was implemented during RNA extraction, following the manufacturer’s protocol. Reverse transcription reactions were carried out using Superscript III reverse transcriptase (Invitrogen), random hexamer primers and 150 ng of total RNA, following the manufacturer’s instructions. Primers for the amplification of *cas9*, *cas1*, and *cas2* gene fragments (Additional file 2: Table S2) were designed to yield PCR amplicons of ~100 bp in size (Fig. 1c). The PCR reaction mixture consisted of 2 μl of cDNA template (150 ng/μl), 0.2 μl of 10-mM dNTP mix, 1 μl of 10-mM forward and reverse oligonucleotide primer, 0.2 μl of Taq DNA polymerase (1 unit), 2 μl of 10× ThermoPol reaction buffer, and 14.6 μl of ddH_2_O, in a total volume of 20 μl. Amplification was carried out at 95 °C for 10 min, followed by 35 cycles of 95 °C for 30 s, 55 °C for 30 s, and 72 °C for 30 s. A final extension step was carried out at 72 °C for 8 min. PCR products were electrophoresed in 2 % agarose gels and visualized with ethidium bromide under UV-light.

Mature crRNAs were PCR amplified using the Quanti-Mir RT Kit (Systems Biosciences) following the manufacturer’s instructions. Briefly, a poly(A)-tail with an attached adaptor sequence was ligated to the mRNA transcripts, and the product was converted to cDNA. crRNAs corresponding to spacers Sp2, Sp3, Sp10, and Sp19 were PCR-amplified from the resulting cDNA library with primers (Additional file 2: Table S2) complementary to the attached adaptor and the individual spacer sequences. The PCR only yielded amplicons from mature crRNAs but not from the unprocessed transcript of the CRISPR array. Each PCR product consisted of a spacer sequence, a flanking part of the direct repeat and an attached poly(A)-tail with a universal primer sequence. The expected size of each of the four tested amplicons is ~85 bp. PCR products were electrophoresed in 2.8 % agarose gels and visualized with ethidium bromide under UV-light.

## Results

### Annotation of the CRISPR-Cas elements and expression *in vitro*

The genome of *Bordetella pseudohinzii* strain 8-296-03 contains three consecutive, apparently co-transcribed, genes (Fig. 1a) that are homologous to *cas9*, *cas1*, and *cas2* of *Alicycliphilus denitrificans* (Additional file 3: Figure S1, Additional file 4: Table S3). Upstream of those genes, a putative tracrRNA is encoded divergently, flanked by a putative promoter and a *rho*-independent stem-loop terminator. Downstream of the *cas* genes, the CRISPR array contains 22 direct repeats (DR) and 21 spacer sequences (Sp). Of these, 19 direct repeats are identical, one repeat (DR-4) has a single nucleotide polymorphism (SNP), and the terminal direct repeat (DR-1) has 3 SNPs (Fig. 1b). While each direct repeat is exactly 36 nucleotides in length, the spacer sequences vary: 19 spacers are 30 nucleotides and two are 29 nucleotides long. The sequence of each spacer is unique. Based on the presence and organization of *cas9*, *cas1,* and *cas2* genes within the operon, we typed this CRISPR-Cas system as Type II-C, according to the classification of the CRISPR-Cas systems established by Makarova *et al.* [6].

A functional CRISPR-Cas system requires expression of the *cas* genes and the CRISPR array, followed by maturation of individual crRNAs. Therefore, we performed an RT-PCR to test whether the *cas* genes are transcribed during growth *in vitro*. Amplicons of *cas9, cas1,* and *cas2* were observed from RNA obtained during both logarithmic and stationary phases of growth (Fig. 1c). Processing of the precursor CRISPR array transcript into mature crRNAs was also confirmed by RT-PCR (Fig. 1d).

To predict putative protospacer targets we submitted each spacer sequence to BLAST search. Table 1 summarizes hits with >80 % hit quality (HQ). Two spacers, Sp8 and Sp9, are identical to prophage elements found in *B. hinzii.* Sp16, Sp10, and Sp20 show high HQ (97 %, 86 %, and 83 %, respectively) with different prophages found in *B. hinzii* and *B. bronchiseptica* and with a capsid gene of a *Microviridae*-family phage (subfamily *Gokushovirinae*), respectively. Spacer Sp13 matches a transposase of the IS3/IS911 family with (90 % HQ). Importantly, several prophages identified as likely sources of spacer elements are not found in the genome of *B. pseudohinzii* 8-296-03 but are present in closely related *B. hinzii*, which appear to lack a CRISPR-Cas system. Collectively, these observations suggest that acquisition of the CRISPR-Cas system by *B. pseudohinzii* conferred CRISPR-mediated protection against these bacteriophages and other mobile genetic elements.

**Table 1:** Highest-scoring BLASTn hits for spacer sequences

BLASTn			Sp#	%ID	%cov	%HQ
Protospacer	Locus tag/name	Organism				
Prophage: hypothetical protein	L544_3238	*Bordetella hinzii* OH87 BAL007II	8	100	100	100
Prophage: intergenic region	L544_1114	*Bordetella hinzii* OH87 BAL007II	9	100	100	100
	L544_1115					
Prophage: replicative DNA helicase	L541_4891	*Bordetella hinzii* CA90 BAL1384	16	100	97	97
	dnaB_2					
Mobile element of IS3/IS911 family	Transposase	*Variovorax paradoxus* EPS	13	90	100	90
Pyruvate carboxyl transferase	C791_4721	*Amycolatopsis azurea* DSM 43854	6	93	97	90
transfer RNA	tRNA-Gly	*Bifidobacterium asteroides* PRL2011	1	93	93	86
Prophage: Siphovirus gp157	BB3535	*Bordetella bronchiseptica* RB50	10	93	93	86
Phage structural component	KF689531.1	*Gokushovirinae* clone BBHD08n1	20	96	86	83
	major capsid					

*Sp#* spacer number, *%ID* percent nt identity, *%cov* percent coverage, *%HQ* percent hit quality

The *S. pyogenes* Cas9 protein (SpyCas9) contains RuvC-like and HNH motifs that were shown to be essential for its function [7]. We searched for both these motifs in the corresponding Cas9 from *B. pseudohinzii* (BpsuCas9) (Additional file 5: Figure S2). The RuvC-like endonuclease motif showed 80 % amino acid (aa) similarity (47 % aa identity) and the HNH motif had 66 % aa similarity (33 % aa identity). In each of the motifs, amino acid residues that are conserved among different type-II Cas9 proteins and that were shown to be essential for Cas9 function are identical in BpsuCas9.

Targeted cleavage by Cas9 requires a protopspacer adjacent motif (PAM), a short sequence, which is 5′-NGG-3′ in *S. pyogenes* [7]. We attempted to determine *in silico* a possible PAM sequence that is recognized by BpsuCas9, but the few available protospacer sequences with a high HQ score (Table 1) limited the number of potential sequence candidates. Although eight predicted protospacers and their flanking sequences are not sufficient to conclusively determine the exact PAM sequence, we propose 5′-WGR-3′ as a potential motif used by BpsuCas9 (Additional file 6: Figure S3).

### Additional *B. pseudohinzii* isolates possess the CRISPR-*cas* locus

**Fig. 2:**
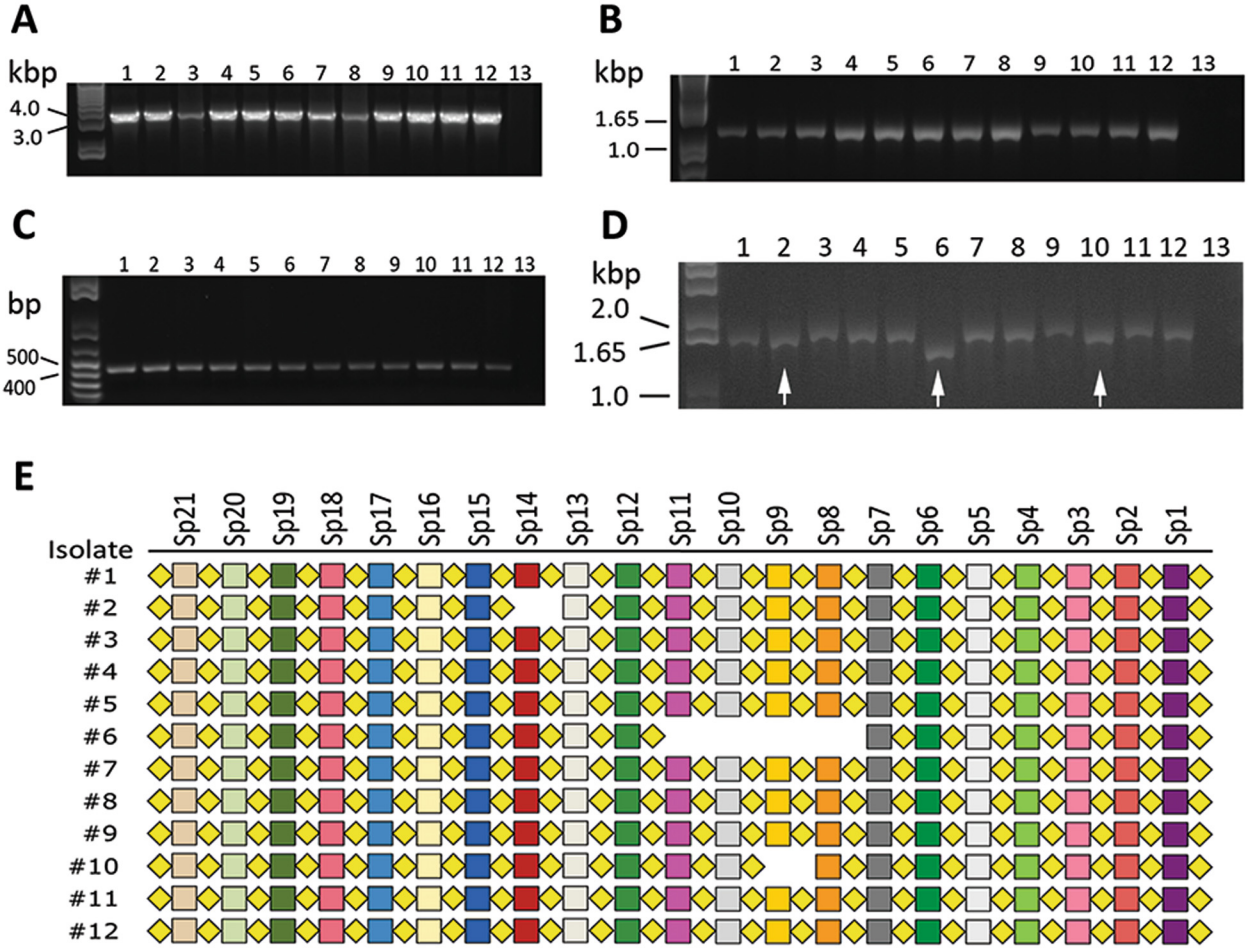
Eleven additional *B. pseudohinzii* isolates possess the CRISPR-*cas* locus. PCR amplicons of *cas9* (**a**), *cas1* (**b**), *cas2* (**c**) and the CRISPR array (**d**) from 12 *B. pseudohinzii* isolates. Lane 1: isolate 8-296-03; lanes 2–12: sequentially obtained isolates #2 to #12, respectively (Additional file 1: Table S1); Lane 13: negative control PCR. **e** Schematic representation of the CRISPR array in each of the isolates. Squares represent spacer sequences. Diamonds represent direct repeats. Arrows in panel D denote isolates that are missing spacer sequences Sp14 (isolate #2), Sp8 to Sp11 (#6) and Sp9 (#10) in panel **e**

Eleven other isolates identified as *B. pseudohinzii* on the basis of their 16S rRNA genes were tested for the presence of a CRISPR-*cas* locus. PCR using gene-specific primers confirmed the presence of *cas9, cas1,* and *cas2* in all isolates (Fig. 2a-c). A CRISPR array was also found in all isolates but some variation in size was observed (Fig. 2d). Sequencing of the CRISPR array PCR amplicons revealed that their lengths are affected by the loss of Sp14 in isolate#2, of Sp8 to Sp11 in isolate#6, and of Sp9 in isolate#10, consistent with the difference in size observed among amplicons (Fig. 2d and e). Each missing spacer is accompanied by the loss of adjacent direct repeat, so that the overall architecture of the array, (−DR-Sp-)_n_, remains intact. The array sequences from all isolates are otherwise identical to one another (Fig. 2e).

### The insertion site of the CRISPR-Cas system is a recombination hotspot in *Bordetella*

**Fig. 3:**
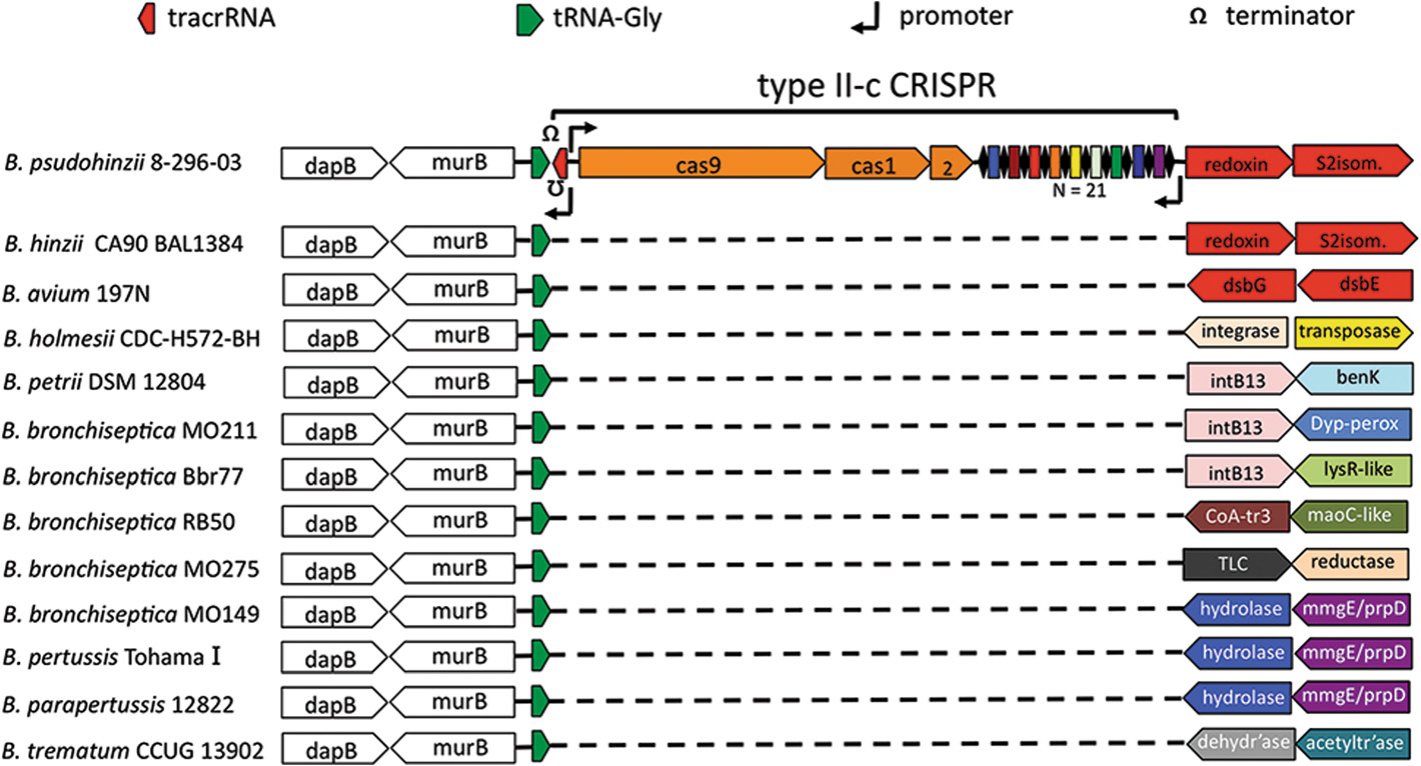
Local gene organization near the insertion site of the *B. pseudohinzii* CRISPR-*cas* locus in multiple *Bordetella* genomes. The genome synteny is conserved upstream the insertions site, while downstream genes vary in both identity and orientation. Dashed lines denote absence of sequence as compared to *B. pseudohinzii* 8-296-03. Genes with the same functional annotations are colored identically. The CRISPR array is shown schematically with black diamonds for direct repeats and colored rectangles for spacer sequences; the total number of spacers is 21

Since we found no evidence for a CRISPR-Cas system in any *Bordetella* species, suggesting it was acquired solely by the *B. pseudohinzii* lineage, we assessed the local gene organization near the insertion site of the CRISPR-*cas* locus in other *Bordetella* genomes (Fig. 3). Only *B. pseudohinzii* and *B. hinzii* exhibit synteny of both the upstream *dapB—murB—tRNA-Gly* gene cluster and the downstream cluster consisting of a reductase (*redoxin*) and a disulfide-isomerase (*S*_*2*_*isom*). In other species, synteny is conserved only upstream of the point at which the CRISPR-Cas locus is located in *B. pseudohinzii*. Genes downstream vary both in identity and orientation, even among isolates of the same species (*B. bronchiseptica*), suggesting this region is a hotspot for recombination.

**Fig. 4:**
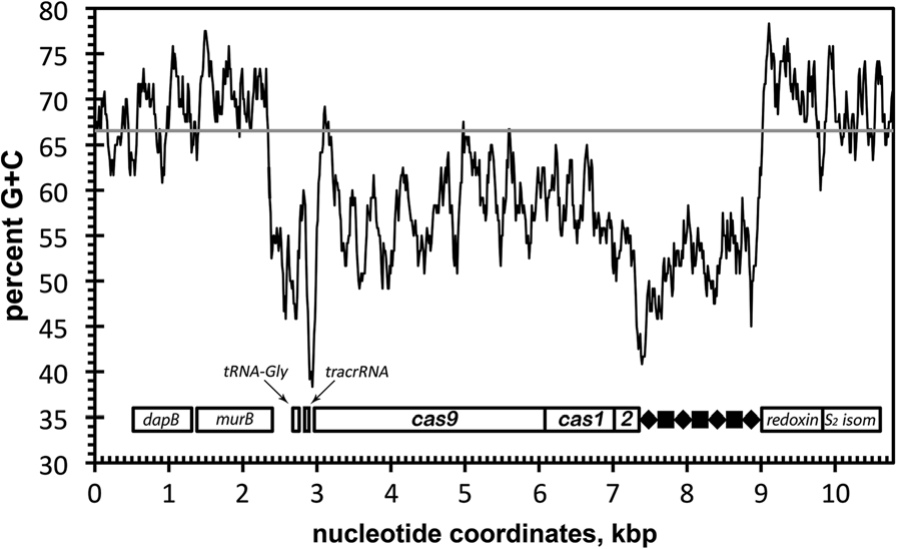
The GC-content of the *B. pseudohinzii* 8-296-03 CRISPR-*cas* locus is significantly lower than that of the genome. The grey horizontal line indicates the average GC-content of 66.5 % for the genome. White rectangles represent genes. The CRISPR array is represented by diamonds (♦, direct repeats) and squares (■, spacer sequences). On the x-axis, 0 corresponds to nucleotide coordinate 24,537 bp of the contig [GenBank:JHEP02000007]

Since absence of the CRISPR-Cas system in the other *Bordetella* species suggests that it was acquired via horizontal gene transfer (HGT), we examined the GC-content of the region including upstream *dapB*—*murB*—*tRNA-Gly* genes, the CRISPR-*cas* locus, and the downstream *redoxin-S*_*2*_*isom* genes. The GC-profile of the upstream and downstream genes is consistent with the genome average of 66.5 % (Fig. 4, grey horizontal line). In contrast, the CRISPR-*cas* locus has a GC-content of 56 %, which is significantly lower (two-tailed Fisher’s exact test, *P* < 0.01). These data strongly suggest that the CRISPR-*cas* locus of *B. pseudohinzii* has been horizontally acquired from an unknown source, likely one with a lower GC-content.

### Evolutionary relationship and horizontal gene transfer

**Fig. 5:**
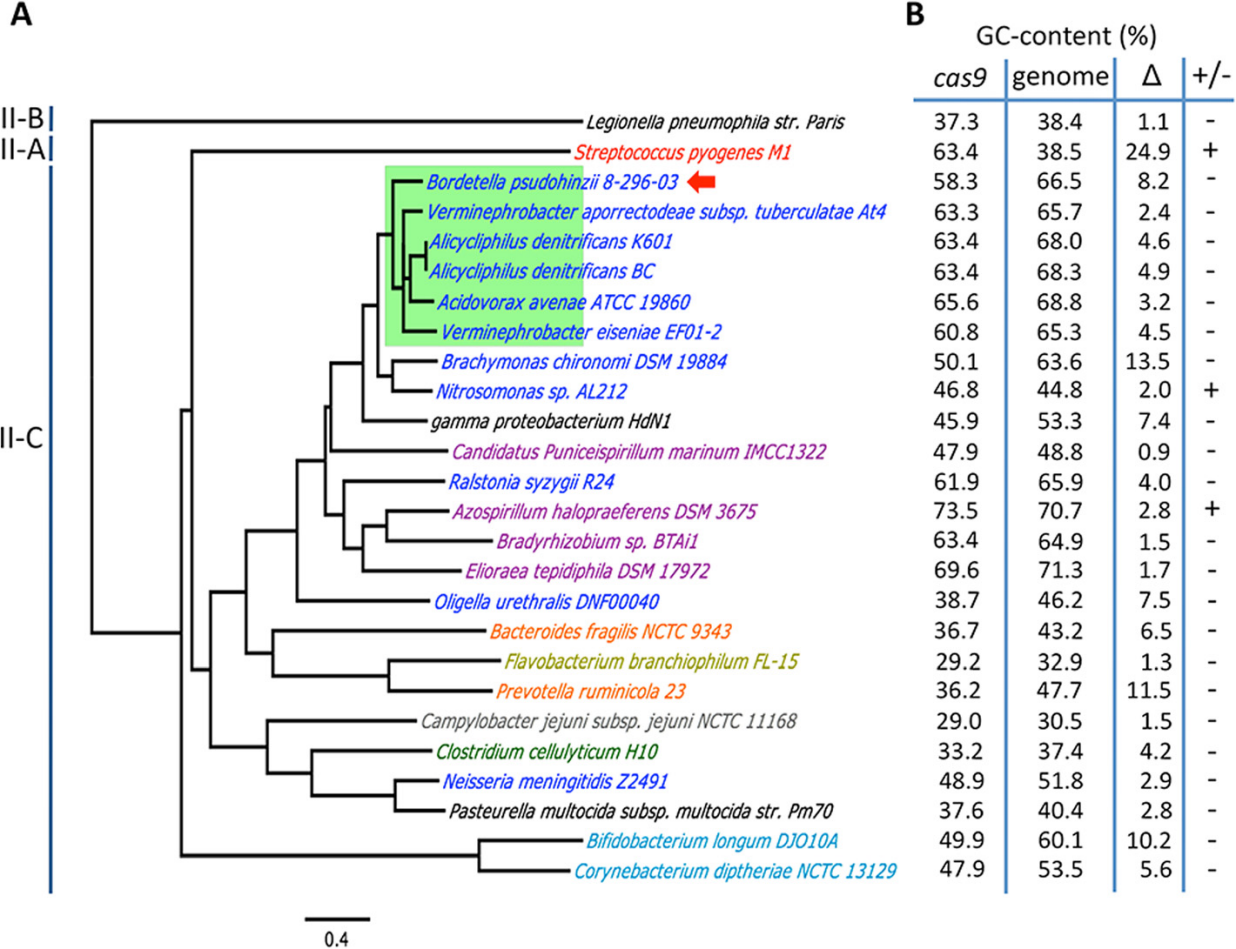
Cas9-based phylogeny and GC-content of *B. pseudohinzii* and the highest-scoring BLAST hits. **a** Maximum likelihood tree based on Cas9 proteins. The green rectangle outlines taxa from the order *Burkholderiales*. Taxa are colored according to their class-level taxonomic assignment: *Gammaproteobacteria* in black, *Bacilli* in red, *Betaproteobacteria* in blue, *Alphaproteobacteria* in purple, *Bacteroidia* in orange, *Flavobacteriia* in golden, *Epsilonproteobacteria* in grey, *Clostridia* in green, *Actinobacteria* in cyan. All nodes have >50 % bootstrap support (10,000 replicates). **b** The GC-content of *cas9* and the corresponding bacterial genome. ∆ is the arithmetic difference between the *cas9* and genome GC-contents; +/− indicates whether *cas9* has a lower (−) or higher (+) GC-content

The Cas9 protein is a signature feature of all type-II CRISPR-Cas systems. To identify a possible source of the *B. pseudohinzii* CRISPR-Cas system, we performed BLAST searches for Cas9 protein sequences (Fig. 5). The two highest-scoring hits, both from *Alicycliphilus denitrificans*, have 74 % aa identity (Additional file 4: Table S3), suggesting that the proposed recent acquisition of this CRISPR-Cas system into the genome of *B. pseudohinzii* was probably from an unknown vector. The Cas9-based phylogeny depicted in Fig. 5 includes the highest-scoring hits together with a subset of selected Cas9 sequences previously published elsewhere [27]. Notably, the 5 closest hits are from related genera, all of which belong to the order *Burkholderiales* in the class *Betaproteobacteria*. Immediately outside of this clade is the Cas9 from other *Betaproteobacteria* and from *gamma proteobacterium* HdN1. The six-member clade of the *Burkholderiales,* including *B. pseudohinzii,* is not the only occurrence of Cas9 in the *Burkholderiales*; *Ralstonia syzygii* and *Oligella urethralis* also belong to this order but possess divergent Cas9 sequences more closely related to those from a variety of *Alphaproteobacteria* (Fig. 5). The presence of closely related bacteria within several clades of the tree suggests multiple, independent HGT events associated with the acquisition of CRISPR systems.

To further explore horizontal acquisition of CRISPR-Cas systems, we calculated the GC-contents for both the *cas9* gene and the genome for all taxa on the tree. The *cas9* sequences ranged from 73.5 % to 29 % GC-content. Likewise, the genomes varied in a similar range from 70.7 % to 30.5 % GC-content. However, in several cases a discrepancy is apparent between the GC-contents of *cas9* and the corresponding genome (Fig. 5b, column ∆). The largest difference was found in *S. pyogenes* M1 whose *cas9* has a GC-content 24.9 % higher than the average for the genome. Discrepancy between the 16S-rRNA-gene tree relating bacterial species and the tree relating their *cas9* gene products suggests horizontal acquisition of the CRISPR-Cas. Similarly, GC-content differences between the CRISPR-*cas* locus and the rest of the genome further support this HGT.

## Discussion

Several lines of evidence suggest that the novel CRISPR-Cas system described here is functional. We observed active transcription of *cas* genes and array sequence, as well as maturation of the array transcript. Further, the array contains multiple spacer sequences with homology to prophages in genomes of the most closely related species, *B. hinzii*. Yet, those prophages are absent from *B. pseudohinzii*, suggesting that the CRISPR-Cas may have provided protection against them as an adaptive immune system.

Interestingly, *B. hinzii* contains prophages and *B. pseudohinzii* contains CRISPR-associated spacer sequences that perfectly match those prophages. These observations indicate that both species have been predated by the same phage and have survived that predation in these two different ways. Acquisition of the prophage or the CRISPR-Cas system, either of which would prevent further phage predation, could have also accelerated the divergent evolution of *B. hinzii* and *B. pseudohinzii* by differently affecting uptake or loss of various other genes, contributing to the observed differences in gene content of these closely related species.

It is often observed that horizontally acquired DNA has a lower GC-content than the genome that receives it [28]; and the GC-content of the CRISPR-Cas system in *B. pseudohinzii* follows this trend. However, our comparison of multiple genomes revealed several cases in which the GC-content of the acquired CRISPR-Cas system is higher than the genome average (Fig. 5). The most striking example is *S. pyogenes* whose *cas9* gene is functional and is successfully used in genome manipulations. This gene has a 25 % higher GC-content than the genome that contains it, suggesting that *S. pyogenes* acquired its CRISPR-Cas system by HGT and that substantial differences in GC-content do not prevent the function of the Cas9 protein.

Recent advances in genome editing, genome engineering, and transcriptional control of genes in multiple organisms take advantage of the endonuclease SpyCas9. However, an important limitation of SpyCas9 is its size. The *S. pyogenes cas9* allele measures 4,107 base pairs, a size that stretches the carrying capacities of some commonly employed vectors. To address this problem, a recent paper described the use of a 3,159-bp gene encoding Cas9 from *Staphylococcus aureus* (SaCas9), which recognizes a different PAM sequence (5′-NNGRR-3′) [29]*.* We introduce BpsuCas9, which is of a similarly small size (3,117 bp) and employs a PAM consensus sequence that putatively consists of 5′-WGR-3′ (Additional file 6: Figure S3) and may provide further flexibility with regards to designing guide RNAs. Future experiments will determine the specific features of the *B. pseudohinzii* CRISPR-Cas system and its potential utility as an additional or alternative tool for genome editing and other applications.

## Conclusions

This study revealed for the first time presence of the CRISPR-Cas system within the genus *Bordetella,* in a genome of newly discovered *B. pseudohinzii* sp. nov. We confirmed that this CRISPR-Cas system is actively transcribed and its crRNAs are processed during bacterial growth. Importantly, the CRISPR array carries spacer sequences matching bacteriophages that infect this and two most closely related *B. hinzii* species, thus, conferring adaptive immunity in *B. pseudohinzii* against these phages. The GC-content analysis of the CRISPR-*cas* locus and homology searches of Cas9 protein sequences explained how single species of *Bordetella* acquired this system horizontally from yet an unknown source. The most important observation made about this *Bordetella* CRISPR-Cas system is its Cas9 endonuclease that is different both in sequence and size from the endonucleases commonly employed in the CRISPR-Cas technology. While the smaller size of BpsuCas9 is of potential utility for more efficient use of biological shuttle vectors during transformations and viral transductions, the unique sequence of BpsuCas9 might allow for some alternative uses of these endonucleases, for example and in addition to the genome editing and genome engineering.

### Availability of supporting data

The data set supporting the results of this article is available in the GenBank repository, [GenBank:JHEP00000000.2] at http://www.ncbi.nlm.nih.gov.

## Additional files

Additional file 1: Table S1. Bacterial isolates used in this study. (DOC 39 kb) Additional file 2: Table S2. Oligonucleotide primer sequences used in this study. (DOC 49 kb) Additional file 3: Figure S1. Top three homologous loci encoding *cas9*, *cas1*, and *cas2*. Top panel, with gene annotations, represents the *cas9-cas1-cas2* locus of *B. pseudohinzii* 8-296-03 (query sequence). Bottom panel summarizes top three BLASTn hit results and illustrates their corresponding alignments against the query. Genome GenBank numbers are shown in blue, above each alignment. (DOC 136 kb) Additional file 4: Table S3. BLASTp comparisons of Type II Cas proteins. (DOC 77 kb) Additional file 5: Figure S2. RuvC-like and HNH-motifs in SpyCas9 and BpsuCas9. RuvC-like motif residue Asp10 and HNH motif residue His840, which are essential for endonuclease activity, are shown in red. Underlined residues are highly conserved among Cas9 proteins from different bacterial species. An * (asterisk) indicates positions at which residue are identical. A: (colon) indicates positions at which residues are of strongly similar properties. A . (period) indicates conservation between residues of weakly similar properties. (DOC 24 kb) Additional file 6: Figure S3. Signatures of protospacer adjacent motif (PAM). Vertical lines denote the same nucleotides and not the base pairing between them. Coloring indicates same nucleotides between predicted target sites. (DOC 62 kb)

## Abbreviations

CRISPR: Clustered regularly interspaced short palindromic repeats
Sp: Spacer sequence
DR: Direct repeat
PAM: Protospacer adjacent motif
crRNA: CRISPR RNA
tracrRNA: Trans-activating CRISPR RNA
HGT: Horizontal gene transfer
SNP: Single-nucleotide polymorphism
GC-content: Guanine + cytosine content
SpyCas9: Cas9 endonuclease of *S. pyogenes*
SaCas9: Cas9 endonuclease of *S. aureus*
BpsuCas9: Cas9 endonuclease of *B. pseudohinzii*
HQ: Hit quality
