# Supplementary material for: A newly discovered Bordetella species carries a transcriptionally active CRISPR-Cas with a small Cas9 endonuclease
Source: BMC Genomics. 2015 Oct 26;16:863. doi: 10.1186/s12864-015-2028-9 (PMC4624362; doi:10.1186/s12864-015-2028-9)
Supplement: Additional file 1: Table S1. — Bacterial isolates used in this study. (DOC 39 kb) [file 12864_2015_2028_MOESM1_ESM.doc]

**Table S1. Bacterial isolates used in this study.**

| Strain | Description | **Source** |
| --- | --- | --- |
| ***Bordetella pseudohinzii*** | | |
| 8-296-03 (isolate#1) | NRRL: B-9942 | K. Boschert, Washington University in St. Louis |
| 8-316-02E (isolate#2) | These isolates were collected sequentially from mice in the same facility over the course of 5 months dating from November 2008 to April 2009. |
| 8-316-03J (isolate#3) |
| 9-040-06A (isolate#4) |
| 9-042-05 (isolate#5) |
| 9-042-14 (isolate#6) |
| 9-090-07 (isolate#7) |
| 9-090-01E (isolate#8) |
| 9-097-01KL (isolate#9) |
| 9-097-03Z (isolate#10) |
| 9-097-01V (isolate#11) |
| 9-111-03E (isolate#12) |
| ***Bordetella hinzii*** | | |
| OH87 BAL007II | NRRL: B59935 | K.B. Register, USDA/ARS/NADC |
| L60 | NRRL: B59936, ATCC51730, LMG14052 |
| DMMZ 1277 | NRRL: B59938 |
| CA90 BAL1384 | NRRL: B59939 |
| 5132 | NRRL: B59940 |
| 4161 | NRRL: B59941 |
