# Supplementary material for: A newly discovered Bordetella species carries a transcriptionally active CRISPR-Cas with a small Cas9 endonuclease
Source: BMC Genomics. 2015 Oct 26;16:863. doi: 10.1186/s12864-015-2028-9 (PMC4624362; doi:10.1186/s12864-015-2028-9)
Supplement: Additional file 2: Table S2. — Oligonucleotide primer sequences used in this study. (DOC 49 kb) [file 12864_2015_2028_MOESM2_ESM.doc]

**Table S2. Oligonucleotide primer sequences used in this study.**

| **Purpose** | **Primer ID** | **Sequence 5' - 3'** | **Direction** | **Target** |
| --- | --- | --- | --- | --- |
| *cas9*, *cas1*, and *cas2* expression assay  (Fig. 1C) | c9_1_F | CGA GAA GAC ATC CAG AAA TG | F | 5' *cas9* |
|  | c9_1_R | CAC CTG TTC GCT CAA TAA C | R | 3' *cas9* |
|  | c1_2_F | TTA CGG CTA CAC CAT TCT | F | 5' *cas1* |
|  | c1_2_R | GTT CCA TGA GAT CGT CAA C | R | 3' *cas1* |
|  | c2_1_F | ATG TGG ATG CTG GTG AT | F | 5' *cas2* |
|  | c2_1_R | CGC AGG TAA ACA CTG AAC | R | 3' *cas2* |
| crRNA detection  (Fig. 1D) | Sp_19F | GTA GCG CGT TAT GTT ACG AAG A | F | Sp19 |
|  | Sp_19R | TCT TCG TAA CAT AAC GCG CTA C | R | Sp19 |
|  | Sp_10F | AAT CCA TGA ACA TTC CCC TCT A | F | Sp10 |
|  | Sp_3F | AGT TTC GTC ACG AGT GGT ACT | F | Sp3 |
|  | Sp_2F | ATT CGG ACT GGC GGA TGT AGC A | F | Sp2 |
|  | Sp_2R | TGC TAC ATC CGC CAG TCC GAA T | R | Sp2 |
| amplification and sequencing of *cas* genes and CRISPR array  (Fig. 2) | cas9-1 | tgc atc ttt ctg ctt gtt ag | F | *cas9* |
|  | cas9-end | TCC TTG ACG ACG AGG AAT C | R | *cas9* |
|  | cas1-1 | GTC GAA ATT GCG GAT GAC | F | *cas1* |
|  | cas1-4 | TAG ATC CAG CAG GGT ATT G | R | *cas1* |
|  | cas2-1 | TCT ACC TGG GAG AAC GAA A | F | *cas2* |
|  | cas2-2 | AGA GTG CTC ATT GTC AGA AA | R | *cas2* |
|  | CRISPR1-5F | GTA CTC CAA TTC ACG GAT AAA | R | array |
|  | CRISPR-endR | ATG ACA ATC AAG GAT CCA GGA C | F | array |
