# Supplementary material for: A newly discovered Bordetella species carries a transcriptionally active CRISPR-Cas with a small Cas9 endonuclease
Source: BMC Genomics. 2015 Oct 26;16:863. doi: 10.1186/s12864-015-2028-9 (PMC4624362; doi:10.1186/s12864-015-2028-9)
Supplement: Additional file 3: Figure S1. — Top three homologous loci encoding cas9, cas1, and cas2. Top panel, with gene annotations, represents the cas9-cas1-cas2 locus of B. pseudohinzii 8-296-03 (query sequence). Bottom panel summarizes top three BLASTn hit results and illustrates their corresponding alignments against the query. Genome GenBank numbers are shown in blue, above each alignment. (DOC 136 kb) [file 12864_2015_2028_MOESM3_ESM.doc]

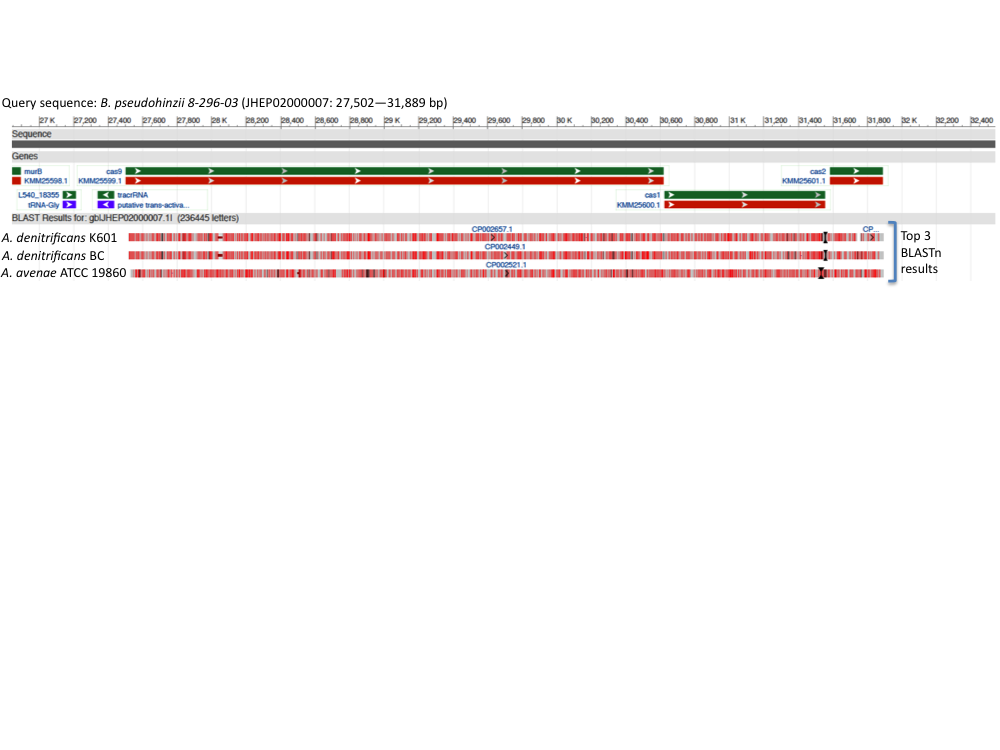


**Figure S1. Top three homologous loci encoding *cas9*, *cas1*, and *cas2*.** Top panel, with gene annotations, represents the *cas9-cas1-cas2* locus of *B. pseudohinzii* 8-296-03 (query sequence). Bottom panel summarizes top three BLASTn hit results and illustrates their corresponding alignments against the query. Genome GenBank numbers are shown in blue, above each alignment.
