# Supplementary material for: A newly discovered Bordetella species carries a transcriptionally active CRISPR-Cas with a small Cas9 endonuclease
Source: BMC Genomics. 2015 Oct 26;16:863. doi: 10.1186/s12864-015-2028-9 (PMC4624362; doi:10.1186/s12864-015-2028-9)
Supplement: Additional file 4: Table S3. — BLASTp comparisons of Type II Cas proteins. (DOC 77 kb) [file 12864_2015_2028_MOESM4_ESM.doc]

**Table S3. BLASTp comparisons of Type II Cas proteins.**

| **Type II CRISPR-Cas-encoding organisms from Figure 5** | **Cas9** | | | **Cas1** | | | **Cas2** | | |
| --- | --- | --- | --- | --- | --- | --- | --- | --- | --- |
|  | **%ID** | **E-value** | **%Cov** | **%ID** | **E-value** | **%Cov** | **%ID** | **E-value** | **%Cov** |
| *Alicycliphilus denitrificans* BC | 74 | 0.0 | 99 | 81 | 8e-175 | 100 | 76 | 3e-57 | 100 |
| *Alicycliphilus denitrificans* K601 | 74 | 0.0 | 99 | 81 | 8e-175 | 100 | 68 | 9e-30 | 70 |
| *Verminephrobacter aporrectodeae* subsp. tuberculatae Al4 | 71 | 0.0 | 99 | 82 | 1e-170 | 94 | 78 | 4e-59 | 100 |
| *Acidovorax avenae subsp. avenae* ATCC 19860 | 71 | 0.0 | 99 | 83 | 9e-174 | 93 | 78 | 6e-59 | 100 |
| *Verminephrobacter eiseniae* EF01-2 | 65 | 0.0 | 100 | 82 | 7e-176 | 94 | 78 | 4e-59 | 100 |
| *Nitrosomonas* sp. AL212 | 62 | 0.0 | 99 | 42 | 2e-06 | 17 | 73 | 1e-54 | 100 |
| *Brachymonas chironomi* DSM 19884 | 60 | 0.0 | 99 | 77 | 4e-175 | 100 | 75 | 2e-55 | 100 |
| *gamma proteobacterium* HdN1 | 55 | 0.0 | 99 | 62 | 3e-127 | 97 | 61 | 6e-45 | 100 |
| *Oligella urethralis* DNF00040 | 40 | 0.0 | 99 | 59 | 1e-115 | 95 | 68 | 1e-45 | 100 |
| *Azospirillum halopraeferens* DSM 3675 | 42 | 0.0 | 99 | 39 | 4e-53 | 85 | 59 | 9e-41 | 100 |
| *Elioraea tepidiphila* DSM 17972 | 39 | 0.0 | 99 | 51 | 4e-89 | 94 | 52 | 3e-32 | 100 |
| *Candidatus Puniceispirillum marinum* IMCC1322 | 39 | 0.0 | 98 | 38 | 3e-67 | 95 | 42 | 3e-24 | 100 |
| *Ralstonia syzygii* R24 | 40 | 0.0 | 99 | 61 | 3e-122 | 98 | 60 | 4e-38 | 98 |
| *Bradyrhizobium* sp. BTAi1 | 39 | 4e-169 | 94 | - | - | - | 39 | 1.6 **^NS^** | 35 |
| *Clostridium cellulolyticum* H10 | 28 | 2e-73 | 81 | 34 | 2e-41 | 81 | 42 | 5e-15 | 85 |
| *Neisseria meningitidis* Z2491 | 29 | 3e-64 | 80 | 34 | 4e-32 | 83 | 38 | 5e-14 | 100 |
| *Pasteurella multocida* subsp. multocida str. Pm70 | 28 | 4e-62 | 80 | 35 | 3e-47 | 77 | 39 | 1e-15 | 100 |
| *Campylobacter jejuni* subsp. jejuni NCTC 11168 | 26 | 9e-60 | 80 | 32 | 4e-29 | 75 | 43 | 8e-17 | 78 |
| *Prevotella ruminicola* 23 | 26 | 2e-55 | 84 | 28 | 0.53^NS^ | 25 | 47 | 3e-24 | 94 |
| *Bacteroides fragilis* NCTC 9343 | 28 | 1e-35 | 64 | 33 | 3e-44 | 88 | 42 | 3e-19 | 100 |
| *Flavobacterium branchiophilum* FL-15 | 33 | 2e-14 | 68 | - | - | - | 47 | 1e-25 | 100 |
| *Corynebacterium diphtheriae* NCTC 13129 | 23 | 6e-11 | 56 | 34 | 1e-36 | 86 | 43 | 8e-25 | 100 |
| *Bifidobacterium longum* DJO10A | 23 | 1e-09 | 56 | 33 | 4e-28 | 74 | 41 | 3e-23 | 100 |
| *Streptococcus pyogenes* M1 GAS | 22 | 3e-09 | 34 | 29 | 3e-28 | 76 | 35 | 1e-12 | 85 |
| *Legionella pneumophila* str. Paris | 25 | 5e-3 | 16 | 28 | 7e-12 | 69 | - | - | - |

%ID – percent aa identity, %cov – percent coverage, NS – non-significant hit.
