# Supplementary material for: A newly discovered Bordetella species carries a transcriptionally active CRISPR-Cas with a small Cas9 endonuclease
Source: BMC Genomics. 2015 Oct 26;16:863. doi: 10.1186/s12864-015-2028-9 (PMC4624362; doi:10.1186/s12864-015-2028-9)
Supplement: Additional file 5: Figure S2. — RuvC-like and HNH-motifs in SpyCas9 and BpsuCas9. RuvC-like motif residue Asp10 and HNH motif residue His840, which are essential for endonuclease activity, are shown in red. Underlined residues are highly conserved among Cas9 proteins from different bacterial species. An * (asterisk) indicates positions at which residue are identical. A: (colon) indicates positions at which residues are of strongly similar properties. A . (period) indicates conservation between residues of weakly similar properties. (DOC 24 kb) [file 12864_2015_2028_MOESM5_ESM.doc]

**A) N-terminal RuvC-like motif**

RuvC-like I

*S. pyogenes* --MDKKYSIGL**D**IGTNSVGWAVITDEYKVPSKKFKVLGNTDRHSIKKNLIGALLFDSGET 58

*B. pseudohinzii* 1 MAKQVRYRLSLDLGSTSLGWAIFR-------------LDADAHPTAIIKAGVRIFSDGRN 47

: :* :.**:*:.*:***:: ::* *. *. :*..*..

**B) HNH-motif**

HNH-motif

*S. pyogenes* LDINRLSDYDVD**H**IVPQSFLKDDSIDNKVLTRSDKNRGKSDNVPSEEVVKKMKN--YWRQ 885

*B. pseudohinzii* ASMLLSEQVEIEHILPFSMTLDDSLNNRTVSLRQANRIKRNRSPWDAREDFEAQGWHYDG 681

.: .: :::**:* *: ***::*:.:: : ** * :. * : . : ::

**Figure S2. RuvC-like and HNH-motifs in SpyCas9 and BpsuCas9.**

RuvC-like motif residue Asp10 and HNH motif residue His840, which are essential for endonuclease activity, are shown in red. Underlined residues are highly conserved among Cas9 proteins from different bacterial species. An * (asterisk) indicates positions at which residue are identical. A : (colon) indicates positions at which residues are of strongly similar properties. A . (period) indicates conservation between residues of weakly similar properties.
