# Supplementary material for: A newly discovered Bordetella species carries a transcriptionally active CRISPR-Cas with a small Cas9 endonuclease
Source: BMC Genomics. 2015 Oct 26;16:863. doi: 10.1186/s12864-015-2028-9 (PMC4624362; doi:10.1186/s12864-015-2028-9)
Supplement: Additional file 6: Figure S3. — Signatures of protospacer adjacent motif (PAM). Vertical lines denote the same nucleotides and not the base pairing between them. Coloring indicates same nucleotides between predicted target sites. (DOC 62 kb) [file 12864_2015_2028_MOESM6_ESM.doc]

Spacer8 5’- CTTCCGGCGGTGCGATCACTTCTTGTTCCA -3’

||||||||||||||||||||||||||||||

OH87 5’- TCTGTTCACCTTCCGGCGGTGCGATCACTTCTTGTTCCATGATTTCC -3’

Spacer9 5’- TATTGGAAGCGAAATCGCGTCGTAACACGT -3’

||||||||||||||||||||||||||||||

OH87 5’- GGTAGGCGGTATTGGAAGCGAAATCGCGTCGTAACACGTTGATTTGC -3’

Spacer9 5’- TATTGGAAGCGAAATCGCGTCGTAACACGT -3’

||||||||||| |||||||||||||||| |

L60 5’- GGTAGGCGGTATTGGAAGCGGAATCGCGTCGTAACACCTTGATTTAC -3’

Spacer16 5’- CTAGTCCCTCAACATGCTGTACTTCGACTT -3’

|||||||||||||||||||||||||||||

BAL1384 5’- CGAGGCCATTAGTCCCTCAACATGCTGTACTTCGACTTAGGCTTCG -3’

Spacer10 5’- CCATGAACATTCCCCTCTACGCCCTCACCC -3’

||||||| || ||||||||||||||||| |

RB50 5’- TGAGGCCCGCCATGAATATGCCCCTCTACGCCCTCACGCAGGAATCA -3’

Spacer10 5’- CCATGAACATTCCCCTCTACGCCCTCACCC -3’

||||||| || ||||||||||||||||| |

S798 5’- TGAGGCCCGCCATGAATATGCCCCTCTACGCCCTCACGCAGGAATAC -3’

Spacer13 5’- GACAGCGGCGGTCGGGAAGGCGTGACGACC -3’

|||| ||||| ||| |||||||||||||||

Variovorax GACGAGGTCGACAACGGCGTTCGAGAAGGCGTGACGACCAGCGAGGC -3’

--------------------------------------------------|||------------

Superimposed consensus PAM for *S. pyogenes* Cas9: NGG

*S. aureus* Cas9: NNGRR

Putative PAM for BpsuCas9: WGR

---------protospacer---------

consensus consensus

**Figure S3. Signatures of protospacer adjacent motif (PAM).**

Vertical lines denote the same nucleotides and not the base pairing between them. Coloring indicates same nucleotides between predicted target sites.
